# The creatine kinase system as a therapeutic target for myocardial ischaemia–reperfusion injury

**DOI:** 10.1042/BST20170504

**Published:** 2018-09-21

**Authors:** Fang Cao, Sevasti Zervou, Craig A. Lygate

**Affiliations:** 1 Division of Cardiovascular Medicine, Radcliffe Department of Medicine and the BHF Centre of Research Excellence, University of Oxford, Oxford, U.K.; 2 Division of Cardiovascular Medicine, Wellcome Centre for Human Genetics, Roosevelt Drive, Oxford OX3 7BN, U.K.

**Keywords:** bioenergetics, cardioprotection, creatine kinase, ischaemia–reperfusion injury

## Abstract

Restoring blood flow following an acute myocardial infarction saves lives, but results in tissue damage due to ischaemia–reperfusion injury (I/R). Ameliorating this damage is a major research goal to improve recovery and reduce subsequent morbidity due to heart failure. Both the ischaemic and reperfusion phases represent crises of cellular energy provision in which the mitochondria play a central role. This mini-review will explore the rationale and therapeutic potential of augmenting the creatine kinase (CK) energy shuttle, which constitutes the primary short-term energy buffer and transport system in the cardiomyocyte. Proof-of-principle data from several transgenic mouse models have demonstrated robust cardioprotection by either raising myocardial creatine levels or by overexpressing specific CK isoforms. The effect on cardiac function, high-energy phosphates and myocardial injury will be discussed and possible directions for future research highlighted. We conclude that the CK system represents a viable target for therapeutic intervention in I/R injury; however, much needed translational studies will require the development of new pharmacological tools.

## Introduction

The energy requirements of the human heart are exceptionally high, requiring the equivalent of 6 kg ATP (adenosine triphosphate) per day [1]. This relies on the continuous recycling of a relatively small ATP pool (∼0.6 g), such that, at any one time, there is only sufficient ATP to power less than 20 s of normal cardiac function [2]. With >90% of ATP generated by mitochondrial oxidative phosphorylation [3], it becomes clear that the onset of ischaemia, and hence anoxia, represents a fundamental problem of energy supply.

Early revascularisation of the ischaemic region has proved to be a highly effective strategy in the clinic, to the extent that mortality from acute myocardial infarction has fallen in recent years, with the correlate that there are now more patients living with the long-term consequences such as heart failure [4,5]. It is well established that the extent of myocardial injury is not just dependent on the ischaemic period, but that a second wave of cellular damage occurs at the point of reperfusion, which can account for up to 50% of the overall injury [6]. Collectively, this has been termed ischaemia/reperfusion (I/R) injury and has been the focus of intense research activity to further improve outcomes.

The cellular conditions that favour reperfusion injury include elevated levels of intracellular calcium, a rising pH (which follows initial acidification during ischaemia) and a burst of reactive oxygen species (ROS) that occurs with reoxygenation [7]. These are all triggers that collectively act to promote opening of the mitochondrial permeability transition pore (mPTP), which leads to a collapse in mitochondrial membrane potential, thereby preventing oxidative phosphorylation and triggering cell death pathways [8,9]. The precise molecular mechanisms and the constituent parts of the mPTP remain a matter of intense debate, which won't be covered here. This review will focus on how cellular energetic status can influence I/R injury and, in particular, the therapeutic potential of enhancing the myocardial creatine kinase system.

## The creatine kinase system in the heart

**Figure 1.**
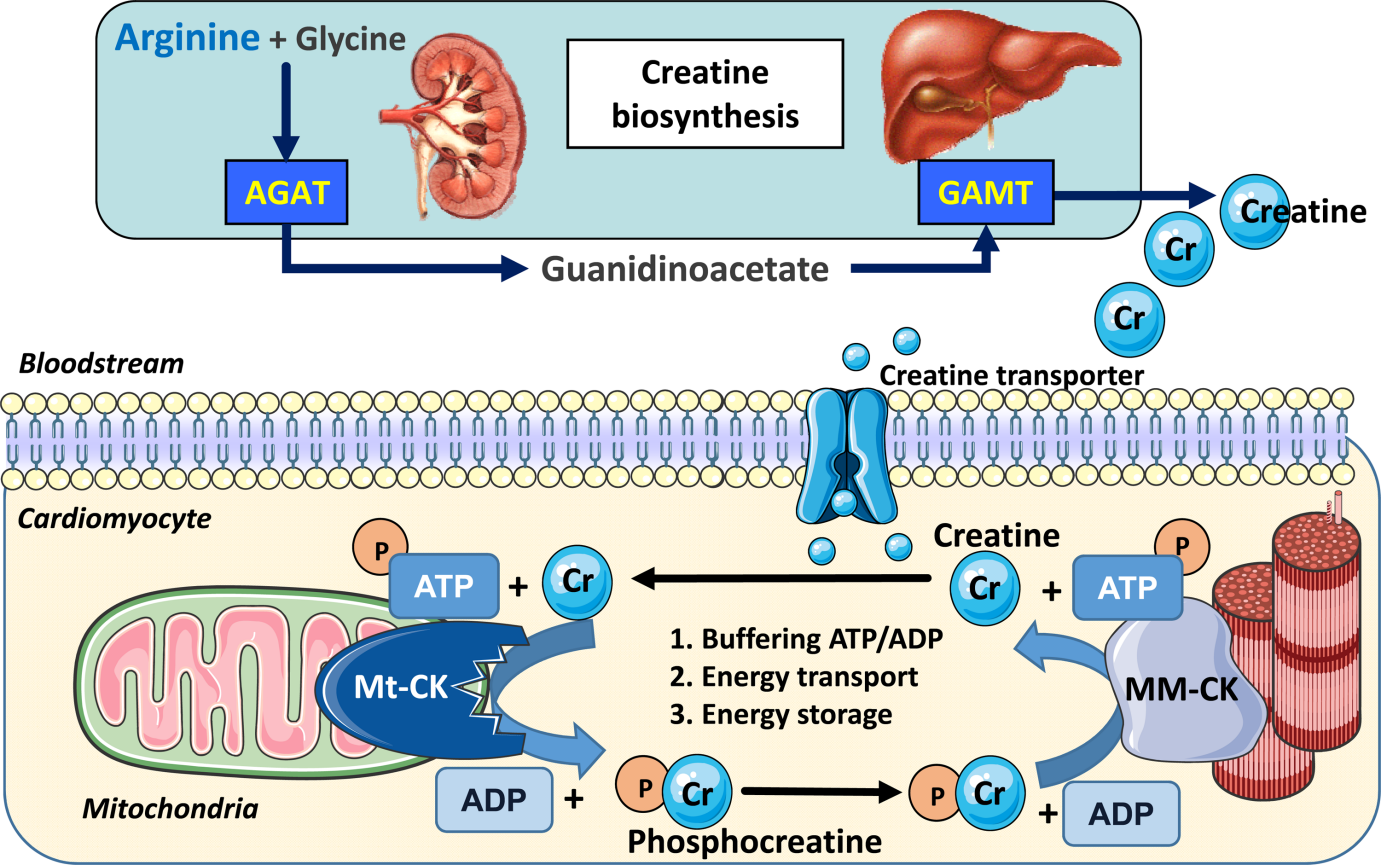
Creatine biosynthesis and the myocardial creatine kinase system. Creatine is a β-amino acid obtained in the diet from animal products or by *de novo* synthesis (∼50%). Arginine-glycine amidinotransferase (AGAT, EC 2.1.4.1) located predominantly in the kidney combines glycine and arginine to form the creatine precursor guanidinoacetate (GAA). GAA is carried in the bloodstream to the liver and pancreas, where it is methylated by guanidinoacetate *N*-methyl transferase (GAMT, EC 2.1.1.2) to form creatine, which is released back into the bloodstream. Uptake into cardiomyocytes is via the specific plasma membrane creatine transporter (SLC6A8), where Mt-CK catalyses the transfer of a phosphoryl group from ATP to form ADP and PCr. PCr accumulates to high levels and is available for the regeneration of ATP at times of high demand catalysed by cytosolic isoforms such as MM-CK. Liberated creatine diffuses back to mitochondria to stimulate further oxidative phosphorylation. Created using Servier Medical Art by Servier which is licensed under a Creative Commons Attribution 3.0 Unported License (http://www.servier.com/slidekit).

Workloads may change dramatically, yet in the normal heart, cellular ATP levels remain static. This is due to the buffering capacity of the creatine kinase (CK) phosphagen system, which involves the reversible transfer of a ‘high-energy’ phosphoryl group catalysed by CK (see Figure 1) [10]:

$$\text{Creatine}+\text{ATP}↔\text{Phosphocreatine}+\text{ADP}+{\text{H}}^{+}$$

The forward reaction occurs within the mitochondrial intermembrane space where the octameric mitochondrial isoform of CK (Mt-CK) is functionally coupled to the adenosine nucleotide translocator (ANT), which channels newly generated ATP, and to the voltage-dependent anion channel (VDAC) [11]. The resultant phosphocreatine (PCr) is relatively less polar and can accumulate to high levels in the cytosol (approximately double that of ATP) [2], where it is highly mobile and available for rapid regeneration of ATP at times of high demand or impaired supply [12]. Thus, PCr represents the primary energy store to buffer ATP levels, the kinetics of which are very fast (∼10 mM/s) [2]. This reverse reaction is catalysed by cytosolic CKs, of which the homodimer muscle isoform is the most abundant (MM-CK) [12], localised primarily at sites of ATP utilisation and allowing functional coupling to ATPases, e.g. myosin ATPase [13] and sarco/endoplasmic reticulum calcium ATPase (SERCA) [14]. There are also very low levels of brain CK forming homodimers (BB-CK) and heterodimers (MB-CK), but the functional significance to the heart is unclear [15].

CK system compartmentalisation is key to its function since this allows for control of local reactant concentrations in energetically favourable ways, i.e. maintaining low ATP/ADP (adenosine diphosphate) levels at the mitochondria to stimulate oxidative phosphorylation and high ATP/ADP levels at the sites of utilisation, which creates thermodynamically favourable conditions to maximise the free energy available from ATP hydrolysis to do work (|Δ*G*_ATP_|) [2,16]. This arrangement also negates the need to rely on relatively slow ATP and ADP diffusion and hence is described as facilitated diffusion [16].

Creatine cannot be synthesised in the cardiomyocyte [17], so must be obtained through the diet, or via *de novo* biosynthesis as shown in Figure 1. Creatine is then actively imported into cardiomyocytes via a specific plasma membrane creatine transporter (CrT; SLC6A8).

**Figure 2.**
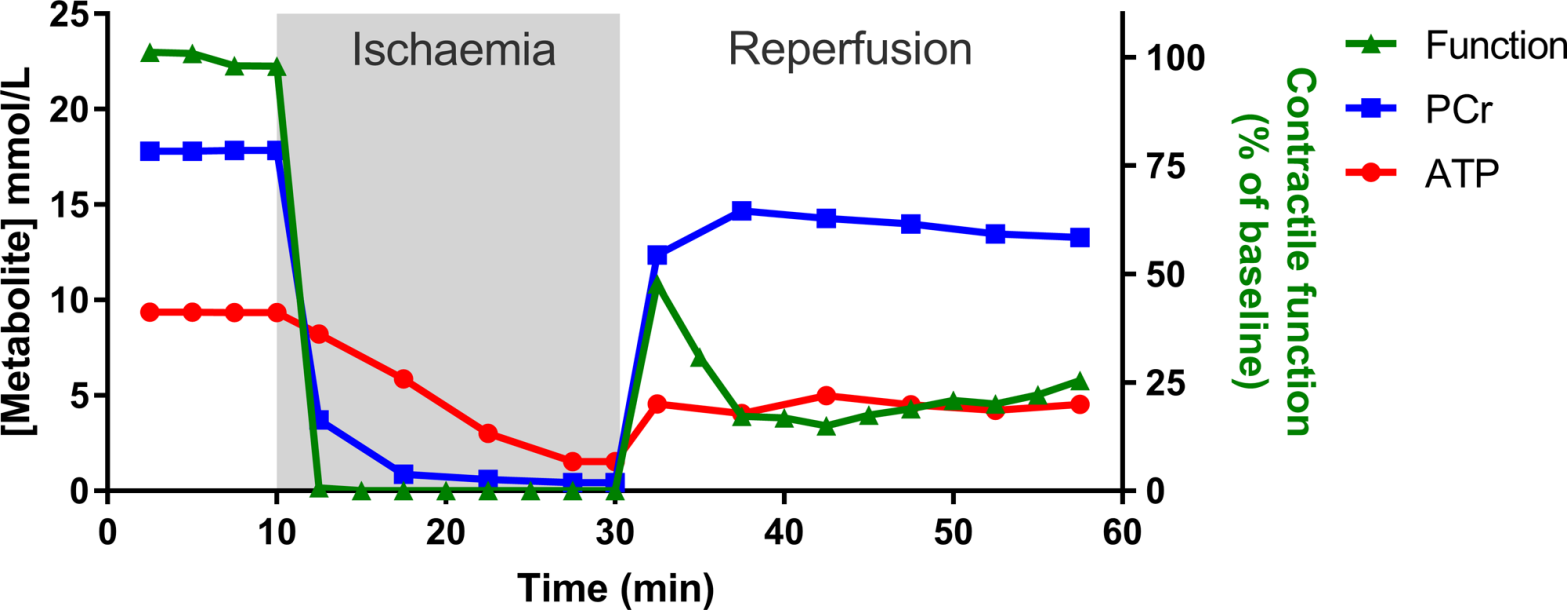
*Ex vivo* measurement of function and high-energy phosphates during no-flow I/R. A classical Langendorff-perfused heart experiment (adapted and redrawn from ref. [49]). The heart is excised and perfused with an oxygenated physiological saline solution, whereupon it beats spontaneously. This can be performed in a magnet to simultaneously measure high-energy phosphates by ^31^P-NMR. After a period of equilibration, the perfusate is switched off to simulate ischaemia. Phosphocreatine (PCr) levels drop within seconds as they are preferentially used to maintain ATP, which only falls once PCr is exhausted. The effect on cardiac contractile function is observed within seconds and quickly falls to zero. ATP continues to reduce slowly during the ischaemic period, dependent on the balance between glycolysis and basal metabolic energy demands (e.g. ionic homeostasis), with accumulation of cellular calcium observed as an increase in end-diastolic pressure. Upon reperfusion, there is a very rapid recovery of PCr, which is critical to re-energising the cell and returning ionic homeostasis before long-term damage is induced. A post-reperfusion spike in function likely reflects high calcium levels, but it is the sustained functional recovery after 30 min reperfusion that is the main outcome measure.

The temporal relationship between cardiac function, ATP and PCr is shown for a classical *ex vivo* I/R experiment in Figure 2. An in-depth analysis with high temporal resolution has indicated that only the phosphorylation potential ([ATP]/[ADP][Pi]) changes more rapidly than contractile function and therefore may represent the limiting metabolic factor [18].

## CK loss of function exacerbates I/R injury

Numerous studies have described the consequences of pharmacological or genetic loss of CK function in the rodent heart. The literature is at times contradictory and has been reviewed in detail elsewhere (see ref. [19]); however, the general consensus is that while loss of CK system components is relatively benign under normal resting conditions, it is detrimental at increased workloads and particularly deleterious in the setting of I/R injury.

The guanidinoacetate methyltransferase (GAMT) knockout (KO) mouse is a case in point. These mice have whole-body creatine deficiency due to an inability to synthesise creatine [20]. When fed a creatine-free diet, KO mice display virtually normal resting cardiac function up to 1 year of age [21,22]. However, they exhibit a reduced contractile reserve, i.e. the ability to increase workload on demand is impaired [22,23]. Crucially, when GAMT KO mouse hearts were subjected to 10 min of global, no-flow ischaemia, followed by 30 min of reperfusion *ex vivo*, the recovery of contractile function was significantly impaired [24% of baseline versus 53% in wild type (WT)] [22]. This was despite compensatory phospho-transfer due to the accumulation of the creatine precursor guanidinoacetate. Left ventricular end-diastolic pressure was also elevated in KO mice by 2.2-fold compared with WT, which is indicative of ischaemic contracture due to excessive calcium accumulation.

A similar picture emerges in mice lacking both M-CK (muscle isoform of creatine kinase) and Mt-CK (mitochondrial creatine kinase; CK double KO), where residual CK activity is ∼3% of WT [24]. The effect on resting *in vivo* cardiac function is subtle and only unmasked using sophisticated imaging techniques [25]. Nevertheless, during 20 min no-flow ischaemia *ex vivo*, end-diastolic pressure rose over 2-fold higher in the KO hearts, which corresponded with greater accumulation of intracellular calcium compared with WT. The consequence was a delayed functional recovery during the reperfusion period [24].

Since M-CK is functionally coupled to SERCA, it is a reasonable hypothesis that CK deficiency may result in an early increase in ADP-to-ATP ratio thereby lowering local |Δ*G*_ATP_| [14]. This would decrease the energy reserve driving SERCA function, thereby affecting calcium resequestration [24,26,27]. Notably, SERCA is particularly susceptible to these effects since it has the highest Δ*G*_ATP_ requirements of all the major ATPases [28].

An important caveat is that these genetic models represent innate loss of CK function, which is likely to result in long-term compensatory adaptations [29], even though these can be difficult to identify [23]. Acute loss of CK function in the adult heart, such as that observed following I/R injury, may therefore have more severe consequences in the absence of such adaptations. Nevertheless, the evidence from chronic loss-of-function studies suggests that an intact CK system is vitally important in responding to I/R injury and that cellular energetics are intrinsically linked to ionic homeostasis and therefore long-term cellular survival.

## CK system gain of function protects against I/R injury

### Treatment with exogenous PCr

The earliest approach to be tested was parenteral administration of PCr at the time of, or shortly before, myocardial ischaemia (for detailed reviews, see refs [30,31]). For example, rats infused with PCr prior to *in vivo* I/R had better contractile function and preserved ATP levels compared with saline controls [32]. Using a similar I/R protocol, Prabhakar et al. [33] found that both PCr and ATP were preserved by prior PCr administration. However, contractile function was not assessed, and in both studies, metabolites were measured in *ex vivo* tissue lysates that could be prone to blood contamination.

PCr is too polar to passively cross the plasma membrane [31] and is not a substrate for uptake via the CrT [34]; therefore, the mechanism by which exogenous administration can alter intracellular energetics remains moot. An alternative explanation is that PCr acts to stabilise membranes by interacting with phospholipids through a mechanism that involves electron charges on both sides of the sarcolemma [30,31,35,36].

Acute PCr administration has been tested in numerous small-scale clinical trials. Although much of the primary literature is in less accessible or non-English language journals, a recent meta-analysis reports overall positive outcomes on short-term all-cause mortality and reperfusion arrhythmias [37]. For example, administration of PCr had anti-arrhythmic properties attributed to membrane stabilisation in open-label, randomised studies of patients with acute myocardial infarction [38] and in patients undergoing elective cardioplegic surgery [39].

### Manipulation of myocardial creatine content

Oral or parenteral administration of creatine has no measurable effect on myocardial creatine content [40], since the heart tightly regulates creatine entry via substrate inhibition of the creatine transporter [41,42]. Genetic or pharmacological interventions are therefore required to force creatine to supra-physiological levels [43].

Our laboratory created a mouse with cardiac-specific overexpression of the creatine transporter (CrT-OE), leading to elevated, but highly variable, levels of myocardial creatine [44]. Counterintuitively, mice with very high creatine (>2-fold, i.e. 140 nmol creatine/mg protein) developed cardiac hypertrophy, dilatation and impaired contractile function, which strongly correlated with intracellular creatine concentrations. One plausible explanation is that CK activity is insufficient to adequately phosphorylate the enlarged creatine pool, resulting in 2-fold higher ADP and reduced |Δ*G*_ATP_|, closely mimicking the impaired bioenergetics observed in heart failure models [44–46]. Further studies, utilising metabolomics and proteomics approaches, showed that excessive creatine has wide-ranging effects on cellular energy metabolism, including reduced glycolytic function [47,48].

This model has allowed us to define a safe range for creatine elevation at 20–100% above normal. Hearts in this moderate range maintain a normal PCr-to-creatine ratio, did not exhibit the detrimental metabolic changes, and exhibited normal cardiac structure and function even at 18 months of age [47,49]. Using an *in vivo* protocol of 45 min no-flow ischaemia and 24 h reperfusion, we found that CrT-OE hearts were protected compared with WT, exhibiting 27% less myocardial necrosis (comparable to ischaemic post-conditioning). In the standard *ex vivo* I/R protocol, the CrT-OE hearts showed a significantly improved recovery of contractile function. ^31^P-NMR (phosphorus nuclear magnetic resonance) revealed that CrT-OE mice had 49% more PCr and higher |Δ*G*_ATP_|, both before onset of ischaemia and very rapidly after reperfusion. This suggests that an expeditious re-energisation of CrT-OE hearts, which if used to regain ionic homeostasis, could underpin the shortened period of reperfusion hyper-contractility [49]. Other mechanisms may contribute, since CrT-OE hearts contained more glycogen, which correlated with creatine levels (*r* = 0.81), and could be used as an anaerobic fuel source during ischaemia. In addition, HL-1 cells loaded with creatine were more resistant to mPTP opening in response to ROS challenge [49]. Many studies have suggested that creatine itself may have a direct antioxidant effect, for example, reducing ROS in skeletal muscle and scavenging superoxide anions and peroxynitrite, but not hydrogen peroxide, in cell-free experiments [50,51]. However, evidence that this represents a significant mechanism in the intact beating heart is currently lacking. Our group has performed ROS challenge studies using hydrogen peroxide and doxorubicin in Langendorff-perfused mouse hearts and found that neither elevating nor depleting myocardial creatine levels influenced outcome [52].

The cardioprotective effect of elevated creatine via CrT-OE appears to be robust since improved functional recovery persists in both males and females, in ageing mice, and in mice with pre-existing cardiac hypertrophy. Furthermore, protection was additive to hypothermic cardioplegia [53].

An alternative strategy has been to partially replace creatine by prior feeding of a synthetic analogue, cyclocreatine, which participates in the CK reaction but at a slower reaction velocity. This has been shown to improve *ex vivo* functional recovery and reduce ischaemic contracture in rat I/R studies since phospho-cyclocreatine persists for longer during the ischaemic period thereby maintaining ATP levels for longer [54–56]. Similar protective effects have been described in rat and dog cardiopulmonary bypass models [57]. The potential downside of this approach is that regeneration of phospho-cyclocreatine is slower than for PCr, which might be limiting at high workloads.

### CK system augmentation

There is substantial evidence in various model systems that augmentation of CK isoforms may also provide protection against I/R injury.

Liver mitochondria from mice overexpressing ubiquitous Mt-CK (an isoenzyme not expressed in heart) were found to be resistant to mPTP opening. This requires localisation of Mt-CK within the intermembrane space and the presence of creatine, which promotes the tight functional coupling between Mt-CK, ANT and VDAC and oxidative phosphorylation [58,59]. Similarly, overexpression of B-CK in the liver led to accumulation of PCr and subsequent protection from hypoxic injury with preservation of ATP over 90 min [60]. It should be noted that liver does not usually express any CK and consequently no PCr [58]; therefore, these studies may represent an exaggerated all-or-nothing response.

The cardiomyocyte-like HL-1 cell line does express CK and is arguably more relevant [61]. When Mt-CK was transiently overexpressed, these cells become more resistant to cell death following hypoxia/reoxygenation challenge [62]. Our laboratory subsequently created a mouse line with cardiac-specific overexpression of Mt-CK. Modest elevation of Mt-CK activity (+27%) did not alter cellular energy metabolism or mitochondrial function, but reduced ischaemic contracture and improved functional recovery upon reperfusion in isolated perfused hearts (58% better than WT). CrT-OE hearts exhibited reduced myocardial infarct size, which was also confirmed *in vivo* (29% versus 55% in WT). Isolated cardiomyocytes from Mt-CK-OE hearts exhibited delayed opening of the mPTP in response to ROS challenge, and this was confirmed independently by measuring mitochondrial swelling following high calcium challenge [63].

A further consideration is that Mt-CK is likely to have a structural role within the mitochondrial membrane space where it forms contact sites that span the inner and outer membranes to provide mechanical stability that may reduce opening probability of the mPTP [11,64–66]. It is notable that ROS promotes the transition from octameric Mt-CK to the dimeric form, which does not form contact sites, is enzymatically less active, and facilitates mPTP opening [64,65,67,68]. This may have consequences since Mt-CK activity closely correlates with recovery of LV function in post-ischaemic myocardium [69].

Conditional overexpression of M-CK in mouse heart is also protective in the *ex vivo* I/R model [70]. At the end of reperfusion, there was significantly improved functional recovery in OE hearts (65% versus 14% in WT) and reduced release of lactate dehydrogenase suggesting less myocardial injury. PCr was not altered at baseline, but recovered to normal levels much more rapidly upon reperfusion, probably reflecting the higher rate of ATP synthesis (CK flux). Rising pH upon reperfusion is known to promote mPTP opening, so it is notable that intracellular pH did not fall as far as WT hearts in the preceding ischaemic phase.

## Future directions of investigation

The current findings from genetically modified mice provide compelling evidence that CK system augmentation provides substantial protection against myocardial I/R injury. However, verification of findings in large animal models is impeded by a lack of pharmacological tools. There are currently no compounds known to up-regulate activity of CK or CrT in the heart, and to our knowledge, no systematic attempts to identify such modulators.

One potential approach may be to circumvent the CrT by giving creatine analogues that can independently cross the plasma membrane. For example, di-acetyl creatine ethyl ester was recently shown to accumulate in brain slices *in vitro* despite CrT inhibition, and although the effect size was modest, this approach deserves further study [71].

Clearly, a greater understanding of how CK and CrT are regulated in health and disease would be useful. By way of example, the only verified endogenous regulator of cardiac CrT is via substrate inhibition (reviewed in ref. [43]). Using an *in vitro* gene array approach, we identified thioredoxin-interacting protein (Txnip) as an endogenous inhibitor of CrT uptake that is switched on when intracellular creatine levels are high [42].

Further study could be directed at whether there is a synergistic effect of simultaneous augmentation of enzyme and substrate. This could easily be accomplished by crossing existing CrT-overexpressing and CK-overexpressing mouse lines. Given that much higher myocardial creatine content may be safe if additional CK is available [49], it is possible that the maximum potential of CK system augmentation in the heart has yet to be realised.

In recent years, there has been much concern at the poor record of translatability from preclinical I/R studies into the clinical setting [72,73]. In accordance with the latest guidelines, the robustness of CK modulation should be tested in co-morbid models, e.g. of diabetes, and should be additive to the protection afforded by P2Y_12_ receptor antagonists [5,74,75]. It is unknown whether CK system augmentation taps into known cardioprotective pathways, such as the reperfusion injury salvage kinases (RISK) pathway [75,76].

Finally, expansion of these findings to other organs at risk from I/R injury, e.g. skeletal muscle, brain, and kidney, is merited, and it remains untested whether this approach would also be beneficial in the transient ischaemia of angina.

## Conclusion

Evidence from genetically modified mice unequivocally shows that an intact CK system is essential for optimal recovery from I/R injury. Furthermore, augmentation of creatine levels or CK activity (regardless of isoform) represents a promising therapeutic strategy that deserves to be further explored.

## Abbreviations

ΔG_ATP_: free energy of ATP hydrolysis
^31^P-NMR: phosphorus nuclear magnetic resonance
ADP: adenosine diphosphate
AGAT: Arginine-glycine amidinotransferase
ANT: adenine nucleotide translocase
ATP: adenosine triphosphate
B-CK: brain isoform of creatine kinase
CK: creatine kinase
CrT: creatine transporter
GAMT: guanidinoacetate N-methyl transferase
I/R: ischaemia / reperfusion
KO: knockout
M-CK: muscle isoform of creatine kinase
mPTP: mitochondrial permeability transition pore
Mt-CK: mitochondrial creatine kinase
OE: overexpressing
PCr: phosphocreatine
ROS: reactive oxygen species
SERCA: sarco/endoplasmic reticulum calcium ATPase
VDAC: voltage-dependent anion channel
WT: wild type
